# Genomic Effects of Biomechanical Loading in Adolescent Human Growth Plate Cartilage: A Pilot Study

**DOI:** 10.1177/19476035241302954

**Published:** 2024-12-10

**Authors:** Zhengpei Zhang, Nageswara Rao Boggavarapu, Laila Sara Arroyo Muhr, Ainhoa Garcia-Serrango, Tim RJ Aeppli, Tobia Sebastiano Nava, Yunhan Zhao, Elena M. Gutierrez-Farewik, Artem Kulachenko, Lars Sävendahl, Farasat Zaman

**Affiliations:** 1 Division of Paediatric Endocrinology, Department of Women’s and Children’s Health, Karolinska Institutet, Solna, Sweden; 2 Division of Obstetrics and Gynaecology, Department of Women’s and Children’s Health, Karolinska University Hospital, Karolinska Institutet, Stockholm, Sweden; 3 Center for Cervical Cancer Elimination, Department of Clinical Science, Intervention and Technology, Karolinska University Hospital, Stockholm, Sweden; 4 KTH MoveAbility Lab, Department of Engineering Mechanics, KTH Royal Institute of Technology, Stockholm, Sweden; 5 Material and Structural Mechanics, Department of Engineering Mechanics, KTH Royal Institute of Technology, Stockholm, Sweden

**Keywords:** genomic, biomechanical loading, human growth plate, RNA-seq, chondrocytes

## Abstract

**Objective:**

The genomic effects of biomechanical loading on human growth plate cartilage are unknown so far. To address this, we used rare human growth plate biopsies obtained from children undergoing epiphysiodesis and exposed them to precisely controlled mechanical loading using a microloading device. The biopsies were cultured 24 hours after mechanical loading, followed by RNA-sequencing analyses to decipher the genomic regulation.

**Design:**

We conducted RNA-seq analysis of human growth plate cartilage obtained from three patients cultured *ex vivo* and subjected to cyclical mechanical loading with peak 0.4 N with frequency 0.77 Hz during a 30-second duration, using a specialized microloading device.

**Results:**

Gene ontology analysis revealed novel data showing three significantly upregulated signaling pathways, including notch, oxytocin, and tight junction, and three significantly downregulated signaling pathways, including lysosome, sphingolipid metabolism, and peroxisome proliferator-activated receptor (PPAR) in human growth plate cartilage. Moreover, we found 15 significantly regulated genes within these signaling pathways from all three patients. These genes included PSEN2, HEY1, and NCOR2 from the notch signaling; CACNB1 and PPP3R2 from the oxytocin signaling; ACTR3C, WHAMM, and ARHGEF18 from the tight junction signaling; ARSA, SMPD1, and CD68 from the lysosome signaling; ARSA and SMPD1 from the sphingolipid metabolism signaling; and SLC27A4 and AQP7 from the PPAR signaling pathway. In addition, 20 significantly upregulated genes and six significantly downregulated genes shared between two patient samples were identified.

**Conclusion:**

Our study provides the first-ever transcriptomic data of mechanical loading of human growth plate cartilage. These findings can potentially provide genetic targets for future investigations in physiological and pathological bone growth conditions.

## Introduction

Longitudinal bone growth, or linear growth, occurs at the growth plate, where chondrocytes proliferate and differentiate into mineralized bone tissue.^1^ The growth plate serves as a center for bone growth and is governed by the delicate interplay of numerous factors, primarily genes, hormones, and mechanical loading.^2^

Mechanical loading, which involves the application of forces on the skeletal system, emerges as a key factor in modulating longitudinal bone growth.^3^ The impact of mechanical loading on longitudinal bone growth depends on the intensity; moderate mechanical loading helps to maintain the integrity of the growth plate, but disuse or overuse can result in growth plate degradation.^4^ Importantly, mechanical loading has been found to increase human bone mineral values,^5^ bone mineral density,^6^ cartilage development and composition,^7^ and skeletal development.^8^ A previous study reported that cyclical mechanical loading with peak force 0.4 N at a low frequency of 0.77 Hz significantly stimulated bone growth in rat embryonic femur bones cultured *ex vivo*.^9^ Furthermore, subjecting one leg in mice to loading of 0.5 N at a high frequency of 20 Hz has also been reported to significantly increase femur length in the loaded leg compared with the contralateral leg.^10^ This growth-promoting effect in the femur was associated with increased growth plate height and number of chondrocytes.

Coordinated proliferation and differentiation of chondrocytes are essential for the continuous elongation of the epiphyseal growth plate. Numerous signaling pathways, including bone morphogenetic protein (BMP) signaling,^11^ transforming growth factor-beta (TGF-β) signaling,^12^ Indian hedgehog (Ihh),^13^ Wnt/ β-catenin signaling pathway,^14^ notch signaling,^15^ peroxisome proliferator-activated receptor (PPAR) signaling,^16^ oxytocin signaling, and insulin-like growth factor 1 (IGF-1)^17,18^ play critical roles in this process. Tight junction signaling is known to help to maintain the chondrocyte layer’s structural and functional integrity. Interestingly, it is also regulated by IGF-1, a key player in the coordination of chondrocyte proliferation and differentiation.^19^ In addition, lysosomal exocytosis participates in the regulation of chondrocyte function and cartilage remodeling within the growth plate.^20^ Furthermore, a recent study has highlighted the significance of sphingolipid metabolism in skeleton development.^21^

Previously, we identified that applying a mechanical loading of 0.4 N at 0.77 Hz significantly enhanced the longitudinal bone growth of embryonic femur bones cultured *ex vivo*.^9^ In this study, we aim to elucidate the gene expression and signaling pathway alterations associated with longitudinal bone growth in human growth plate cartilage subjected to the same mechanical loading. Currently, there are no data on the impact of mechanical loading on signaling pathways and genes involved in longitudinal bone growth within human growth plate cartilage. This study aims to address this gap in knowledge and will contribute to a deeper understanding of the connection between mechanical loading and the regulation of genes involved in longitudinal bone growth.

## Material and Methods

### Culture of Human Growth Plate Biopsies

The collection of human growth plate biopsies was approved by the local human ethics committee (Karolinska Institutet Research Ethics Committee North at the Karolinska University Hospital, Stockholm, Sweden). Informed consent was obtained from all subjects and their parents, which was documented in the original hospital records. We collected the growth plate biopsies from the distal femur and proximal tibial growth plates simultaneously in adolescent patients with familial tall stature (two girls, one boy; age range: 12-15 years) undergoing epiphysiodesis surgery. This is a condition characterized by an individual growing significantly taller than average without any underlying medical disorders affecting growth. Immediately after collection, the biopsies were sliced and cultured in Dulbecco’s modified Eagle’s medium/nutrient mixture F-12 Ham (DMEM-F12) medium containing gentamycin (10 μg/ml), 0.2% bovine serum albumin (BSA), 1 mM beta-glycerophosphate, and ascorbic acid (50 μg/ml), as previously described.^22^

### Application of Mechanical Loading on Human Growth Plates

As previously described, we used an easy-to-operate microloading device capable of applying dynamic mechanical loading via a controller and frame.^9^ The design of this device facilitates loading in both *ex vivo* cultured bones and *in vivo* conditions while minimizing the risk of sample damage, as the force control is verified in the software accompanying the controller. Before applying mechanical load, the microloading device is clamped to a table and connected to the laptop controller and router. This small mobile device can be used at room temperature.

**Figure 1.**
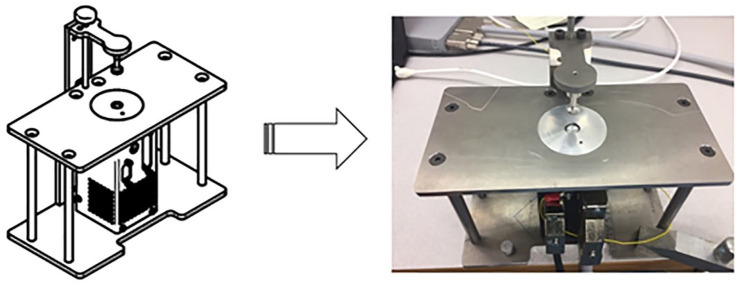
Customized microloading device from sketch to reality. The microloading device can specifically deliver repetitive loading to *ex vivo* cultured tissue.^9^

The main components of the device include a linear actuator, actuator controller, wireless broadband router, and mounting frame and computer. The loading instruments consist of an indenter with a convex elastomer cover, a flat indenter, and 3D-printed needle attachments. The device can apply forces ranging from 0.05 to 5 N, with repetitive impacts (30-180 reps) and continuous sinusoidal loading (5-20 Hz). In addition, the adjustable tool height (min. 80 mm) improves sample positioning, further reducing the risk of damage^9^ (**Fig. 1**).

The force control was verified using the controller’s software, with the first cycle dedicated to adjustments to ensure the maximum force was not exceeded. Subsequent load cycles were repeatable and accurately followed the target profile while maintaining a low level of noise. The pressure range achieved through mechanical loading at various levels was confirmed using pressure-sensitive film, which indicated that color intensity on the contact surface increased with load. This suggests that the 0.1 MPa threshold is surpassed at approximately 0.5 N. Mechanical loading of 0.4 N at 0.77 Hz was used in this study, as it was identified to significantly enhanced the longitudinal bone growth of embryonic femur bones cultured *ex vivo*.

**Figure 2.**
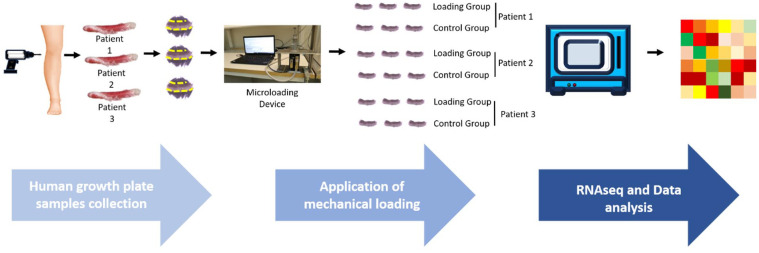
Experimental setup for studying mechanical loading effects on human growth plate cartilage. Briefly, human growth plate tissue samples collected from children after epiphysiodesis surgeries were carefully isolated from bone structures. These samples were then cut into small pieces (marked with yellow stripe lines) and randomly assigned into two groups: one group was subjected to one-time mechanical loading in a computer-controlled micro-mechanical loader and another one (control group) was sham-loaded. The biopsies were thereafter cultured for 24 hours followed by RNA sequencing.

Fresh growth plate biopsies were under light microscopy excised into small pieces, which were immediately transferred to a Petri dish containing culture medium. Thereafter, six biopsies (one biopsy per well in a 24-well plate) from each patient were randomly allocated into two groups: the mechanical loading group (*n* = 3) and the control group (*n* = 3), with biopsies from all three patients included in both groups. Each biopsy fragment from the single patient was treated as an individual observation, as each piece undergoes independent exposure to mechanical loading and is cultured separately, including RNA extraction. This methodology mirrors that of fetal rat embryonic metatarsal bones derived from the same embryos, where each bone is treated as a distinct observation due to separate treatment.^23^ Before applying mechanical loading, the growth plate tissue specimen was vertically positioned on the indenter to transmit the force laterally through the growth plate. In the loading group, we administered mechanical loading at 0.4 N with 0.77 Hz over 30 seconds. This regimen, as previously established in our research, has been shown to facilitate bone growth and was applied using our customized microloading device.^9^ In the control group, we followed the same procedure, but no mechanical loading was applied. Mechanical loading was immediately applied to the biopsies after their random allocation into the 24-well plate (**Fig. 2**).

### RNA Preparation From Human Growth Plate Biopsies Exposed to Mechanical Loading

After 24 hours of culture, RNA was extracted from loaded and unloaded biopsies using the Quick-RNATM Microprep Kit (Zymo Research, USA), according to the manufacturers’ instructions. Briefly, each biopsy was lysed by adding 300 μl lysis buffer to a cryotube containing ZR Bashing beads (Zymo Research) and homogenized using the Disruptor Genie (Zymo Research). Each biopsy was then incubated with DNase I (5 μl 1U/μl DNase mixed with 35 μl DNA digestion buffer) at room temperature for 15 minutes and washed with 400 μl RNA prep buffer, 700 μl RNA wash buffer and another 400 μl RNA wash buffer; 15 μl DNase/RNase-free water was added, so that, RNA from each biopsy was extracted and stored at −80 °C until further use.

### RNA-seq Library Preparation and Analysis

RNA libraries for next-generation sequencing were constructed individually from all the samples using the well-established Smart-seq2 protocol. A total of 1 ng of total RNA was subjected to reverse transcription.^24^ Tagmentation was performed in all individual cDNA libraries using the Nextera XT kit (Illumina Inc., USA), using dual index barcodes (IDT technologies, Belgium).^25^ The final amplified libraries were purified with Ampure XP beads (Beckman Coulter, USA) in a ratio of 1:1.

All individual libraries were validated, normalized, pooled, denatured, and diluted. The pool was sequenced paired-end (151 + 151 cycles), using the Illumina Novaseq 6000 (Novogene [UK] company limited). The processing and analysis of raw sequencing data were performed using the Partek^®^ Flow^®^ platform (Partek Inc., USA). Briefly, Illumina adapters were trimmed from all the sequencing reads, and the adaptor-trimmed sequencing reads were aligned to the human genome GRCh38 (hg38) using the STAR aligner with default parameters. Reads were then quantified against GENCODE human genes release 35. Mitochondrial, ribosomal, and genes detected with expression levels < 1 in at least 80% of the samples were filtered out.

### Statistical Analysis

Differential analysis was performed between the groups using the DESeq2 tool, considering genes and effects as statistically significant when they had a *P* value < 0.05 and a fold change (FC) threshold of ≤ −2 or ≥ 2. Pathway analysis was conducted using the KEGG database, and pathways with a *p* value < 0.05 were considered significant biological signaling pathways.

## Results

### Significantly Regulated Genes by Mechanical Loading in Individual Patients

**Figure 3.**
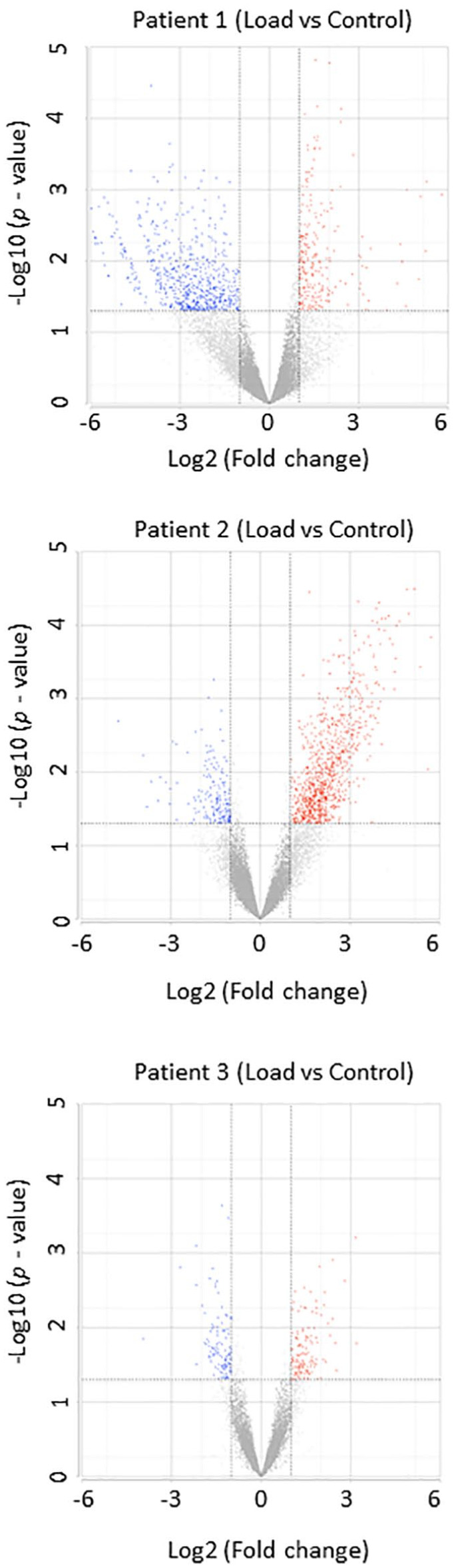
Volcano plot-based profiling of mechanically regulated genes in individual patients. The red dots denote upregulated genes, the blue dots denote downregulated genes, and the black dots denote non-significant genes.

We identified notable genes that responded to mechanical loading in three patients. Patient 1 exhibited 250 genes with significant upregulation and 685 genes showing downregulation. For patient 2, we observed 980 genes with significant upregulation and 174 genes with downregulation. Regarding patient 3, 134 genes upregulated significantly and 111 genes downregulated significantly (**Fig. 3**).

### Significantly Regulated Genes Shared Among Individual Patients

**Figure 4.**
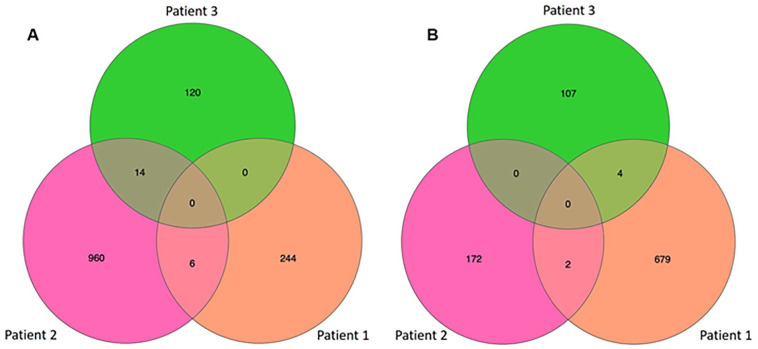
Venn diagram illustrating significantly **(A)** upregulated and **(B)** downregulated common genes upon mechanical loading shared between each patient.

A Venn diagram illustrates upregulated (**Fig. 4A**) and downregulated genes (**Fig. 4B**) shared across three individual patients. Among the upregulated genes, six genes, including activating transcription factor 3 (ATF3), FP236383.4, FP236383.5, FP236383.6, mitochondrially encoded tRNA glutamic acid (MT-TE), and period circadian regulator (PER1) are shared between patients 1 and 2, and 14 genes, including AP001437.2, dynein axonemal heavy chain 10 (DNAH10), EMC3 antisense RNA 1 (EMC3-AS1), LINC01503, mitochondrial methionyl-tRNA formyltransferase (MTFMT), NK2 homeobox 5(NKX2-5), SEC14 like binding 3 (SEC14L3), tumor protein translationally controlled 1 pseudogene 8 (TPT1P8), tubulin tyrosine ligase-like 3 (TTLL3), VAC14 antisense RNA 1 (VAC14-AS1), Z99129.3, zinc finger protein 66 (ZNF66), zinc finger protein 99 (ZNF90), and zinc finger protein 555 (ZNF555) are shared between patients 2 and 3 **(Tables 1 and 2).** However, no upregulated genes are shared between patients 1 and 3. Regarding the downregulated genes, two genes, including aquaporin 9 (AQP9) and stathmin 1 (STMN1) are shared between patients 1 and 2, 4 genes, including AC107208.1, family with sequence similarity 66, member C (FAM66C), harbinger transposase-derived 1 (HARBI1), and ring finger protein, transmembrane 2 (RNFT2) are shared between patients 1 and 3 **(Tables 3 and 4).** There are no shared downregulated genes between patients 2 and 3. In addition, no upregulated or downregulated genes are shared across all three patients.

**Table 1.** A List of Six Significantly Upregulated Genes in the Human Growth Plate Cartilage of Both Patients 1 and 2.

Gene Name	Patient 1 Fold Change	Patient 1 *P* value	Patient 2 Fold Change	Patient 2 *P* Value
ATF3	2.722	0.015	3.311	0.041
FP236383.4	2.009	0.004	3.436	0.026
FP236383.5	2.187	0.001	3.554	0.021
FP671120.6	2.156	0.002	3.552	0.021
MT-TE	2.708	0.013	5.205	0.021
PER1	2.467	0.045	3.929	0.027

**Table 2.** A List of 14 Significantly Upregulated Genes in the Human Growth Plate Cartilage of Both Patients 2 and 3.

Gene Name	Patient 2 Fold Change	Patient 2 *P* value	Patient 3 Fold Change	Patient 3 *P* Value
AP001437.2	4.785	0.039	2.161	0.041
DNAH10	5.944	0.006	2.402	0.036
EMC3-AS1	3.134	0.041	2.057	0.027
LINC01503	4.112	0.038	3.629	0.006
MTFMT	4.470	0.014	2.922	0.039
NKX2-5	9.461	0.012	2.448	0.039
SEC14L3	4.412	0.017	2.627	0.018
TPT1P8	6.753	0.018	2.359	0.045
TTLL3	3.108	0.029	2.125	0.005
VAC14-AS1	4.206	0.031	2.000	0.032
Z99129.3	8.395	0.008	2.249	0.014
ZNF66	3.014	0.047	2.033	0.030
ZNF90	6.706	0.005	2.050	0.005
ZNF555	2.833	0.014	2.050	0.019

**Table 3.** A List of Two Significantly Downregulated Genes in the Human Growth Plate Cartilage of Both Patients 1 and 2.

Gene Name	Patient 1 Fold Change	Patient 1 *P* value	Patient 2 Fold Change	Patient 2 *P* Value
AQP9	−4.646	0.031	−3.014	0.014
STMN1	−2.319	0.014	−2.018	0.037

**Table 4.** A List of Four Significantly Downregulated Genes in the Human Growth Plate Cartilage of Both Patients 1 and 3.

Gene Name	Patient 1 Fold Change	Patient 1 *P* value	Patient 3 Fold Change	Patient 3 *P* Value
AC107208.1	−10.900	0.016	−2.296	0.020
FAM66C	−9.090	0.013	−2.068	0.024
HARBI1	−13.219	0.010	−2.227	0.031
RNFT2	−9.197	0.013	−2.274	0.034

### Identification of Signaling Pathways and Genes Affected by Mechanical Loading

**Figure 5.**
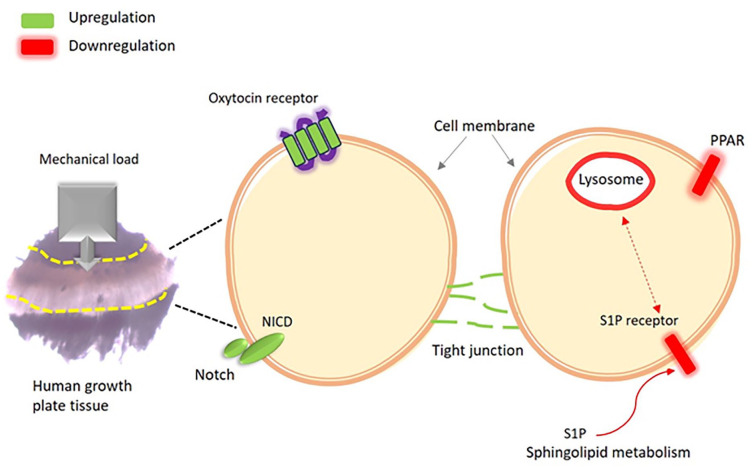
Overview of chondrocyte signaling pathways significantly regulated upon mechanical loading (0.4 N, 0.77 Hz, 30 seconds) of cultured human growth plate cartilage in three patients.

**Figure 6.**
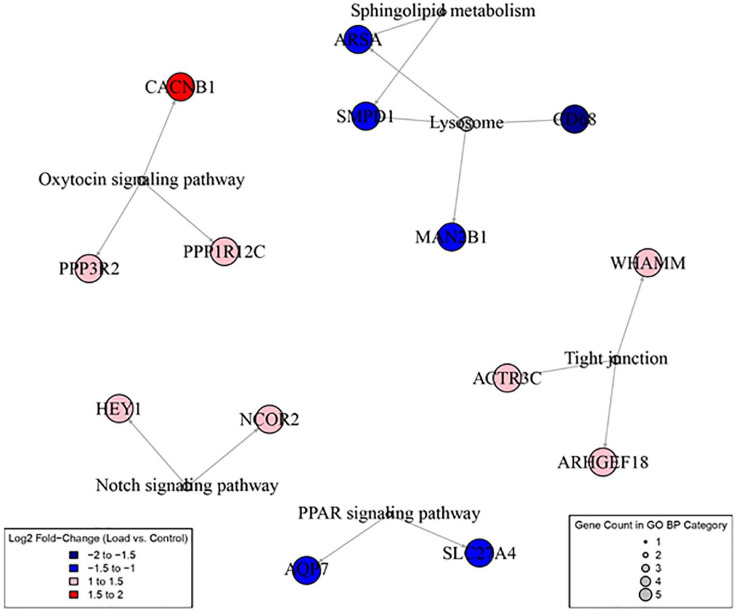
Neural graph network displaying the six signaling pathways that were detected as significantly regulated, along with the genes within these pathways that were significantly affected by mechanical loading (0.4 N at 0.77 Hz, 30 seconds) in cultured human growth plate cartilage across three patients.

Analysis of all samples together (load group *n* = 9, control group *n* = 9, replicates) revealed a total of six distinct signaling pathways significantly affected by mechanical loading. Our data revealed significant regulatory patterns within these pathways, with three pathways showing significant upregulation: notch (*P* = 0.003), oxytocin (*P* = 0.039), and tight junction signaling pathways (*P* = 0.043) (**Table 5**). Conversely, three pathways displayed downregulation: lysosome (*P* = 0.003), sphingolipid metabolism (*P* = 0.023), and PPAR signaling pathways (*P* = 0.042) (**Table 6**). An overview of these six signaling pathways significantly regulated by mechanical loading is shown in **Figure 5**. The neural network analysis also highlights genes within these six signaling pathways affected significantly by mechanical loading (**Fig. 6**). Neural network figures included all signaling pathways, both significant and non-significant, showing downregulation (**Supplementary Fig. 1A, B**), and upregulation (**Supplementary Fig. 2A, B**).

**Table 5.** Significantly Upregulated Signaling Pathways in Cultured Human Growth Plate Cartilage (*n* = 3 From Each of Three Patients) Treated With Mechanical Force 0.4 N at 0.77 Hz Over 30 Seconds.

Description	Enrichment Score	*P* Value
Notch signaling pathway	3.401	0.003
Oxytocin signaling pathway	3.257	0.039
Tight junction signaling pathway	3.140	0.043

**Table 6.** Significantly Downregulated Signaling Pathways in Human Growth Plate Cartilage (*n* = 9) Exposed to Mechanical Loading 0.4 N at 0.77 Hz Over 30 Seconds.

Description	Enrichment Score	*P* Value
Lysosome signaling pathway	5.844	0.003
Sphingolipid metabolism signaling pathway	3.746	0.023
PPAR signaling pathway	3.172	0.042

**Figure 7.**
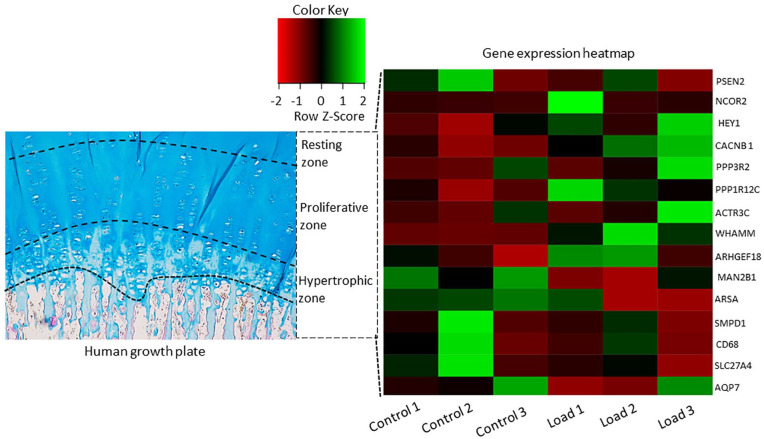
Heatmap illustrating genes within the significantly upregulated and downregulated signaling pathway affected by mechanical loading (0.4 N at 0.77 Hz, 30 seconds) among cultured human growth plate cartilage in three patients.

In addition, we identified a total of 15 significantly regulated genes from the signaling pathways in the loaded growth plate group compared with the untreated group. These include PSEN2 (presenilin-2), HEY1 (hairy/enhancer-of-split related to YRPW motif protein 1), and NCOR2 (human nuclear receptor co-repressor 2) from the notch signaling pathway; CACNB1(calcium voltage-gated channel auxiliary subunit beta 1), and PPP3R2 (protein phosphatase 3 regulatory subunit B, beta), PPP1R12C (protein phosphatase 1 regulatory subunit 12C) from the oxytocin signaling pathway; ACTR3C (actin-related protein 2/3 complex), WHAMM (WASP homolog associated with actin, Golgi membranes and microtubules), and ARHGEF18 (Rho/Rac guanine nucleotide exchange factor 18) from the tight junction signaling pathway; MAN2B1 (mannosidase alpha class 2B member 1), ARSA (arylsulfatase A), SMPD1 (sphingomyelin phosphodiesterase 1), and CD68 (cluster differentiation 68) from the lysosome signaling pathway; ARSA and SMPD1 from sphingolipid metabolism signaling pathway; and SLC27A4 (solute carrier family 27 member 4) and AQP7 (aquaporin-7) from the PPAR signaling pathway (**Fig. 7**).

## Discussion

### Significantly Upregulated Genes Shared Across Two Patients

Six upregulated genes, including ATF3, FP236383.4, FP236383.5, FP236383.6, MT-TE, and PER1 are found to be shared across patients 1 and 2. ATF3 has been shown to be directly responsive to mechanical loading,^26^ PER1 could influence the adaption to mechanical loading through circadian regulation of physiological processes^27,28^ and MT-TE could support the energy demands during mechanical loading process via its role in mitochondrial function.^29,30^ Collectively, these genes could help chondrocytes to respond and manage mechanical loading, maintaining cartilage health and function. However, there are no current data regarding the regulation of mechanical loading on genes FP236383.4, FP236383.5, and FP236383.6.

Fourteen upregulated genes, including AP001437.2, DNAH10, EMC3-AS1, LINC01503, MTFMT, NKX2-5, SEC14L3, TPT1P8, TTLL3, VAC14-AS1, Z99129.3, ZNF66, ZNF90, and ZNF555, are found to be shared across patients 2 and 3. NKX2-5 is known to be regulated by mechanical loading in bone and heart tissues.^31,32^ In this study, we identified that NKX2-5 is also regulated by mechanical loading in cartilage tissue. AP001437.2, EMC3-AS1, LINC01503, and VAC14-AS are noncoding RNAs, which are known to modulate chondrocyte differentiation, proliferation, and extracellular matrix production.^33^ Importantly, MTFMT may be upregulated to meet the increased energy demands due to heightened mitochondrial activity required during mechanical loading.^34,35^ Structural genes, such as DNAH10 and TTLL3, are essential for maintaining cytoskeletal integrity and intracellular transport, which is crucial for chondrocyte function under mechanical stimuli.^36,37^ SEC14L3 may influence lipid signaling and membrane dynamics, which is important for maintaining cell membrane stability and signaling under mechanical loading.^38,39^ ZNF66, ZNF90, and ZNF555 are zinc finger proteins, which are known to be regulated by mechanical loading, ensuring proper cellular adaption.^40,41^ However, the roles of TPT1P8 and Z99129.3 remain largely unexplored.

### Significantly Downregulated Genes Shared Across Two Patients

Two downregulated genes, including AQP9 and STMN1, are shared between patients 2 and 3. AQP9 can facilitate water and solute transport to manage osmotic balance and hydration,^42^ while STMN1 modulates microtubule dynamics to maintain cytoskeletal integrity.^43^ Together, these adjustments support cartilage maintenance, function, and enable chondrocytes to adapt to mechanical loading.

Four downregulated genes, including AC107208.1, FAM66C, HARBI1, and RNFT2, are shared between patients 1 and 3. FAM66C, as a noncoding RNA, could regulate chondrocyte differentiation, proliferation, and extracellular matrix production.^33^ However, there are no specific data regarding the regulation of mechanical loading on HARBI1, RNFT2, and AC107208.1.

It is noteworthy that we observed commonly shared upregulated or downregulated genes only between two patients, rather than among all three patients. This occurrence is attributed to instances where a gene is found upregulated in one or two patients but downregulated in the other patient(s).

### Signaling Pathways Activated by Mechanical Loading

#### Notch signaling pathway

Our data showed that the notch signaling pathway was significantly upregulated (*P* = 0.003) in human growth plate cartilage exposed to mechanical loading (**Table 5**).

The notch signaling pathway is a cell-to-cell communication pathway involved in various cellular and tissue processes in skeletal development and homeostasis.^44^ Mechanical loading has previously been linked to the notch signaling pathway. For example, decreased mechanical loading was reported to reduce notch-1 signaling in the mandibular condylar cartilage of growing rabbits.^45^ Throughout chondrogenesis, notch signaling has been demonstrated to have context-dependent functions.^46^ Specifically, notch signaling inhibits early chondrogenic cell differentiation via SOX9 and proliferation independent of RBPJk; however, it stimulates mesenchymal stem cells by inhibiting their differentiation and hypertrophy.^46-48^

Recent studies have indicated a beneficial effect of activating notch signaling in bone.^49^ Activation of notch in osteocytes can initiate a beneficial bone-building response in mature bones.^50^ Likewise, disruptions in notch signaling can lead to skeletal abnormalities and growth disorders, such as spondylocostal dysostosis and short-trunk dwarfism characterized by impaired vertebrae and ribs.^51^ In our study, we identified three genes within the notch signaling pathway: PSEN2, a feedback regulator on notch, exhibited notable downregulation. Conversely, NCOR2 and HEY1, both serving as transcription factors in the notch signaling pathway, demonstrated significant gene upregulation in human growth plate cartilage exposed to mechanical loading (**Fig. 7**). These findings highlight the potential relevance of these gene targets for further investigation into the role of the notch signaling pathway in bone growth modulation through mechanical loading.

#### Oxytocin signaling pathway

Our analysis revealed a notable upregulation (*P* = 0.039) of the oxytocin signaling pathway in human growth plate cartilage upon exposure to mechanical loading (**Table 5**).

Emerging studies have investigated the role of oxytocin in cartilage and chondrocyte biology. Chondrocytes have been found to express oxytocin receptors in animals and humans, suggesting their responsiveness to oxytocin.^52,53^ The presence of these receptors suggests that oxytocin may directly influence chondrocyte function. Indeed, previous reports have shown that oxytocin treatment promotes chondrocyte proliferation, suggesting a role in the regulation of cartilage growth and repair.^53^ In addition, oxytocin has been shown to enhance the differentiation of chondrocytes, leading to the production of cartilage-specific extracellular matrix components.^52^

In animal models of cartilage injury or osteoarthritis, oxytocin treatment has been reported to promote cartilage repair and reduce degenerative changes. Furthermore, oxytocin has been reported to stimulate the synthesis of cartilage matrix molecules and enhance the regenerative capacity of chondrocytes.^54^ It is well-known that chondrocytes can release inflammatory mediators, contributing to cartilage inflammation and degradation in conditions, such as osteoarthritis.^55^ Oxytocin has been found to exert anti-inflammatory effects on chondrocytes by suppressing the production of pro-inflammatory molecules.^56^ This anti-inflammatory action of oxytocin may help mitigate the inflammatory processes associated with cartilage damage.

In addition, a previous study in mice showed bone anabolic effects of oxytocin, including increased bone formation and improved bone microarchitecture.^57^ Similarly, a study in rats reported that intraperitoneal injection of oxytocin increased osteoprotegerin levels and bone remodeling, indicating a beneficial effect of oxytocin on bone formation.^58^

Besides the significant activation of the oxytocin signaling pathway in response to mechanical loading, we identified genes, such as CACNB1, PPP3R2, and PPP1R12C, which respond to changes in intercellular calcium levels, indirectly interact with the oxytocin signaling pathway and are upregulated in response to mechanical loading (**Fig. 7**). Collectively, our findings establish a connection between mechanical loading and the oxytocin signaling pathway in human growth plate cartilage. Considering the known influence of mechanical loading on bone growth,^9,10^ our data present a plausible mechanism involving the oxytocin signaling pathway. Furthermore, there is potential speculation that pivotal genes within the oxytocin signaling pathway could serve as viable targets, thereby shedding light on the impact of mechanical loading on the development of longitudinal bone growth.

#### Tight junction signaling pathway

Our findings indicate a significant upregulation (*P* = 0.043) (**Table 5**) of the tight junction signaling pathway in human growth plate cartilage after exposure to mechanical loading. Tight junctions are intercellular adhesion complexes in epithelia and endothelia that define tissue spaces and control the selective movement of solutes across the epithelium.^59^ Studies have demonstrated that mechanical loading can modulate the arrangement and organization of tight junction proteins, resulting in changes in barrier permeability.^60^ For example, cyclic stretching of epithelial cells has been observed to increase the permeability of the tight junctions, potentially affecting the transport of molecules across the tissue.^61^ Furthermore, mechanical loading can activate intracellular signaling pathways that modulate tight junction integrity. These pathways involve various molecules, including mechanosensitive proteins and cytoskeletal components. For instance, the activation of Rho GTPases, which are involved in cytoskeletal organization, can impact tight junction assembly and function of tight junction in response to mechanical cues.^62^

Mechanical loading can induce tissue remodeling processes, such as cell migration and tissue repair. Tight junctions may undergo dynamic changes during these processes to facilitate cell movement and tissue restructuring. For example, shock waves which regulate mechanotransduction, not only open tight junctions,^60^ but also prevent vismodegib-induced growth retardation.^63^ Similarly, in epithelial wound healing, the rearrangement of tight junction proteins allows for cell migration and resealing of the epithelial barrier.^64^

Dysregulation of tight junctions is associated with various diseases, including inflammatory bowel diseases,^65^ cancer,^66^ and cardiovascular disorders.^67^ However, few studies have investigated whether mechanical loading can regulate skeletal development through tight junctions. A transcriptomic analysis in small and large breed dogs reported height-related differences in the tight junction pathway, linking this pathway to skeletal development.^68^ In addition, claudin, known as tight junction proteins, is a family of proteins suggested to be involved in bone remodeling.^69^ Furthermore, bone loss was reported in claudin-18 knockout mice, which further linked the tight junction signaling pathway to skeletal development.^70^

In alignment with our observed upregulation of the tight junction, ACTR3C, WHAMM, and ARHGEF18, which are regulators of cytoskeletal dynamics, also exhibited significant upregulation upon mechanical loading (**Fig. 7**). Collectively, these findings contribute valuable insights into the effect of mechanical loading on human growth plate cartilage.

### Signaling Pathways Suppressed by Mechanical Loading

#### Lysosome signaling pathway

Our data revealed a notable downregulation (*P* = 0.003) (**Table 6**) of the lysosome signaling pathway within human growth plate cartilage following exposure to mechanical loading. Lysosomes are membrane-bound organelles found within cells involved in the degradation and recycling of cellular waste, including proteins, lipids, and organelles.^71^ Mechanical loading can affect the generation and accumulation of these waste products^72^, which, in turn, may influence lysosomal function and cellular homeostasis.

It is known that lysosomal exocytosis is a repair mechanism for patching ruptured membranes, for example, under conditions of increased biomechanical load.^73^ Following membrane disruption, the F-actin network undergoes depolymerization due to a rapid equilibration of intracellular Ca^2+^, triggering lysosome accumulation and lysosomal fusion to reseal the perforation. This process is essential for cells to survive in mechanically active environments.^74-78^ During skeletal development, it has also been demonstrated that destructive hydrolases are released via lysosomal exocytosis from hypertrophic growth plate chondrocytes.^20^

While understanding the specific effects of mechanical loading on lysosomes is still evolving, we made a novel finding that mechanical loading significantly suppressed the lysosome signaling pathway within human growth plate cartilage. Furthermore, our investigation identified a cluster of genes within the lysosome signaling pathway that is downregulated in response to mechanical loading. This cluster includes MAN2B1, ARSA, SMPD1, and CD68 (**Fig. 7**). Altogether, our findings in human growth plate cartilage support earlier data suggesting a link between the lysosome signaling pathway and mechanical loading.

#### Sphingolipid metabolism signaling pathway

We also found that sphingolipid metabolism was significantly downregulated (*P* = 0.023) (**Table 6**) in human growth plate cartilage after being exposed to mechanical loading.

Sphingolipid metabolism refers to the biochemical pathways involved in the synthesis, degradation, and interconversion of sphingolipids.^79^ One particular sphingolipid, ceramides, has been linked to the regulation of longitudinal bone growth.^21,80^ Specifically, C2-ceramide is suggested to inhibit proliferation and induce apoptosis in growth plate chondrocytes.^80^

Mechanical loading has been shown to induce ceramide production and the subsequent activation of stress-responsive signaling pathways, such as apoptosis and inflammation.^81^ The level of pro-apoptotic ceramides can be influenced by mechanical loading-induced changes in sphingolipid metabolism, leading to the activation of cell death pathways.^81^ Conversely, mechanical loading can also affect the production of anti-apoptotic sphingolipids, such as sphingosine-1-phosphate, which may promote cell survival.^82^ Sphingolipids have also been implicated in tissue remodeling processes in which mechanical loading plays a crucial role.^83^ In the skeleton system, the sphingomyelin phosphodiesterase 3 (SMPD3) is a crucial factor in sphingolipid metabolism.^21^ Interestingly, a study has found that GW4869-mediated inhibition of SMPD3 function can accelerate the mineralization of chondrogenic ATDC5 cultures, suggesting that sphingolipids may contribute to chondrocyte activities.^84^

While the specific effects of mechanical loading on sphingolipid metabolism are not yet fully understood, our study presents novel insights: several genes within the sphingolipid metabolism pathway, including ARSA and SMPD1 (**Fig. 7**), exhibited downregulation following the exposure of human growth plate chondrocytes to mechanical loading. Notably, chondrocytes express cerebroside-sulfatase (also known as ARSA), a factor involved in sulfatide degradation within the extracellular matrix.^85,86^ In addition, phospholipase C contributes to chondrocyte proliferation.^87^ Nonetheless, further investigation is needed to elucidate the precise implications of these genes within the interactions between sphingolipid metabolism and loading in chondrocytes.

#### PPAR signaling pathway

We observed PPAR signaling pathway undergoes a significant downregulation (*P* = 0.042) (**Table 6**) within human growth plate cartilage following exposure to mechanical loading.

The PPAR signaling is a crucial regulatory pathway involved in various cellular processes, including inflammation^88^ where it has been reported to have anti-inflammatory properties and can regulate the expression of inflammatory genes.^89^ Mechanical loading can also impact inflammatory processes, and PPARs may play a role in this modulation. Indeed, PPARγ is expressed in growth plate chondrocytes, and the activation of PPAR promotes adipogenic trans-differentiation of growth plate chondrocytes, suggesting that the PPAR signaling pathway is critical for chondrocytes and bone growth.^16,90^

Furthermore, we found that SLC27A4 and AQP7 were downregulated (**Fig. 7**). Within chondrocytes, SLC27A4 plays a pivotal role in regulating the metabolism of fatty acids, which are integral constituents of cell membranes and energy storage.^91^ Significantly, fatty acids assume crucial functions in modulating the expression of specific genes in chondrocytes, thereby influencing their activities and contributing to cartilage homeostasis.^92^ AQP7 serves to facilitate the transport of glycerol across cell membranes and glycerol, in turn, exerts influence over diverse cellular processes in chondrocytes, including extracellular matrix synthesis, cellular signaling, and the maintenance of cartilage homeostasis.^93,94^

Briefly, our investigation has revealed significant inhibition of the PPAR signaling pathway by mechanical loading, along with the downregulation of the genes SLC27A4 and AQP7. These findings provide insights into the involvement of the PPAR signaling pathway within human growth plate cartilage. Further studies are imperative to investigate the specific roles of the PPAR signaling pathway concerning the impact of different levels of mechanical loading in human growth plate cartilage.

### Physiological and Pathological Loading

Physiological loading on the growth plate involves the application of mechanical forces that mimic natural levels of physical activity. These forces vary based on body weight, movement type, and tissue condition and are crucial for bone growth. In humans, evidence of physiological loading is seen in professional tennis players, who exhibit wider bones in their playing-side arms.^95^ Specifically, the playing-side ulna and second metacarpal were found to be 3% and 3.7% longer, respectively, compared with the contralateral side. Control subjects, on the other hand, showed no measurable differences in bone length between their dominant and non-dominant arms. Another study reported that mechanical loading applied to mice at a frequency of 20 Hz and 0.5 N significantly increased femur length.^10^ In juvenile mice, 100 compressive loading cycles at 5 N and a frequency of 2 Hz (mimicking ambulation) accelerated longitudinal bone growth.^96^ Similarly, the loading regime applied (0.4 N at a frequency of 0.77 Hz) in the present study has previously been shown to increase bone growth in rat embryonic femur bones cultured *ex vivo*.^9^

In contrast, mechanical loading with excessive force (pathological condition) can have detrimental effects, such as knee osteoarthritis associated with obesity.^97,98^ A study in rats showed that applying an excessive force of 17 N at a frequency of 2 Hz resulted in a 4% reduction in ulna length.^99^ Mechanistically, excessive mechanical forces have been reported to trigger cell death and collagen damage in bovine cartilage explants subjected to a single impact load of 15-20 megapascals (MPa).^100^ Similarly, in canine cartilage explant, loading of 5 MPa at 0.3 Hz has been shown to cause collagen damage and chondrocyte necrosis and apoptosis.^101,102^

### Limitations

There are several limitations in our study. First, the number of growth plate samples used in this study is low, and the RNA extracted from the whole growth plate, including resting, proliferating, and hypertrophic chondrocytes is small due to their scarce availability. Consequently, this scarcity hinders our capacity to validate our findings through additional functional experiments. Due to the limited availability of growth plate biopsies, we combined samples from the distal femur and proximal tibia. While we randomized the allocation of these samples into control and loading groups to balance potential site-specific effects, we acknowledge that inherent anatomical and biomechanical differences between these sites could have influenced gene expression responses. This could potentially contribute to the variability observed in our results. Future studies with larger sample sizes should consider analyzing these sites separately to account for anatomical variations. Second, these biopsies were collected from adolescents (two girls and one boy) of varying ages (12-15 years). The growth plates from these tall-stature-adolescents, defined as a height of more than 2 standard deviation above average for same sex of age,^103^ may exhibit differences in growth plate biology, stages of puberty, and genetic background. These factors could explain why commonly shared upregulated and downregulated genes were identified in only two patients rather than all three.

Moreover, children with constitutional tall stature generally show normal length at birth but experience accelerated growth velocity in early childhood.^104^ Their growth trajectory runs parallel to, but above, standard growth curves, and they continue growing along this higher percentile until puberty.^105,106^ While recent studies suggest that increased expression of the growth hormone receptor gene may contribute to their distinctive growth pattern,^107^ limited research has explored the underlying biology of their growth plates. In our study, deviations from typical growth trajectories in these adolescents could affect how their growth plates respond to mechanical load in *ex vivo* studies. Finally, we have conducted tests solely on a single magnitude of mechanical loading, primarily due to the scarcity of this tissue. Nevertheless, these unique and rare human growth plate tissues offer us an opportunity to enhance our understanding of the genomic effects.

## Conclusion

Our analysis has revealed noteworthy differential regulation of signaling pathways in response to mechanical loading in human growth plate cartilage, never reported before. Specifically, three signaling pathways, such as notch, oxytocin, and tight junction, displayed significant upregulation, while another three signaling pathways, namely lysosome, sphingolipid metabolism, and PPAR, exhibited marked downregulation. Furthermore, 15 significantly regulated genes were found within these six signaling pathways. Notably, we also identified upregulated and downregulated common genes shared across two patients, which have never been reported for their association with mechanical loading in human growth plate cartilage. These novel genetic insights contribute to an enhanced understanding of transcriptomic response to mechanical loading in human growth plate cartilage. Overall, these findings hold promise in illuminating the intricate interplay between mechanical stimuli and growth plate cartilage, potentially advancing our comprehension of longitudinal bone growth under pathological and non-pathological conditions.

## Supplemental Material

sj-pdf-1-car-10.1177_19476035241302954 – Supplemental material for Genomic Effects of Biomechanical Loading in Adolescent Human Growth Plate Cartilage: A Pilot Study Supplemental material, sj-pdf-1-car-10.1177_19476035241302954 for Genomic Effects of Biomechanical Loading in Adolescent Human Growth Plate Cartilage: A Pilot Study by Zhengpei Zhang, Nageswara Rao Boggavarapu, Laila Sara Arroyo Muhr, Ainhoa Garcia-Serrango, Tim RJ Aeppli, Tobia Sebastiano Nava, Yunhan Zhao, Elena M. Gutierrez-Farewik, Artem Kulachenko, Lars Sävendahl and Farasat Zaman in CARTILAGE
