# Supplementary material for: Genomic Effects of Biomechanical Loading in Adolescent Human Growth Plate Cartilage: A Pilot Study
Source: Cartilage. 2024 Dec 10;17(3):829–44. doi: 10.1177/19476035241302954 (PMC11629350; doi:10.1177/19476035241302954)
Supplement: Supplementary file 1 [file sj-pdf-1-car-10.1177_19476035241302954.pdf]

(A)

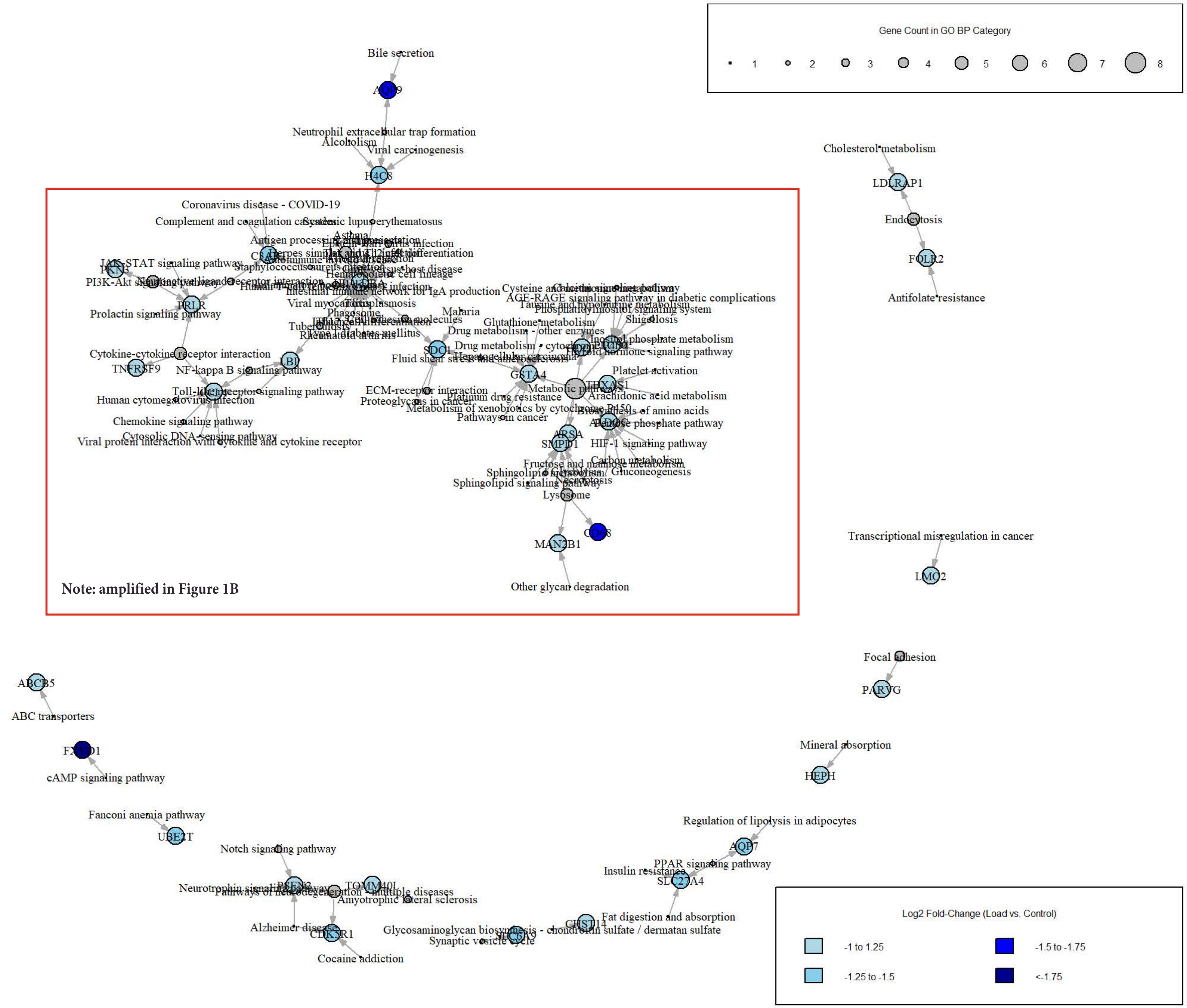

Gene Count in GO BP Category
1
2
3
4
5
6
7
8
Log2 Fold-Change (Load vs. Control)
-1 to 1.25
-1.25 to -1.5
-1.5 to -1.75
<-1.75
Bile secretion
Neutrophil extracellular trap formation
Alcoholism
Viral carcinogenesis
H4C8
Coronavirus disease - COVID-19
Complement and coagulation cascades
Systemic lupus erythematosus
JAK-STAT signaling pathway
PRLR
Prolactin signaling pathway
Cytokine-cytokine receptor interaction
TNFRSF9
NF-kappa B signaling pathway
LBP
Toll-like receptor signaling pathway
Human cytomegalovirus infection
Chemokine signaling pathway
Cytosolic DNA-sensing pathway
Viral protein interaction with cytokine and cytokine receptor
Intestinal immune network for IgA production
Viral myocarditis
Toxoplasmosis
Phagosome
Tuberculosis
Type I diabetes mellitus
Rheumatoid arthritis
Malaria
SDC1
Fluid shear stress and atherosclerosis
ECM-receptor interaction
Proteoglycans in cancer
Cysteine and methionine metabolism
AGE-RAGE signaling pathway in diabetic complications
Taurine and hypotaurine metabolism
Phosphatidylinositol signaling system
Shigellosis
Glutathione metabolism
Drug metabolism - other enzymes
Inositol phosphate metabolism
Thyroid hormone signaling pathway
Hepatocellular carcinoma
GSTA4
Platelet activation
Metabolic pathways
Arachidonic acid metabolism
Platinum drug resistance
Metabolism of xenobiotics by cytochrome P450
Biosynthesis of amino acids
Pathways in cancer
Pentose phosphate pathway
ARSA
SNPID1
HIF-1 signaling pathway
Carbon metabolism
Glycolysis / Gluconeogenesis
Sphingolipid signaling pathway
Lysosome
MAN2B1
Other glycan degradation
Note: amplified in Figure 1B
Cholesterol metabolism
LDLRAP1
Endocytosis
FOLR2
Antifolate resistance
Transcriptional misregulation in cancer
LMO2
Focal adhesion
PARVG
Mineral absorption
HEPH
Regulation of lipolysis in adipocytes
AQP7
PPAR signaling pathway
Insulin resistance
SLC27A4
Fat digestion and absorption
CHST14
Glycosaminoglycan biosynthesis - chondroitin sulfate / dermatan sulfate
Synaptic vesicle cycle
ABCB5
ABC transporters
FXYD1
cAMP signaling pathway
Fanconi anemia pathway
UBE2T
Notch signaling pathway
TOMM40L
Amyotrophic lateral sclerosis
Alzheimer disease
CDK5R1
Cocaine addiction

(B)

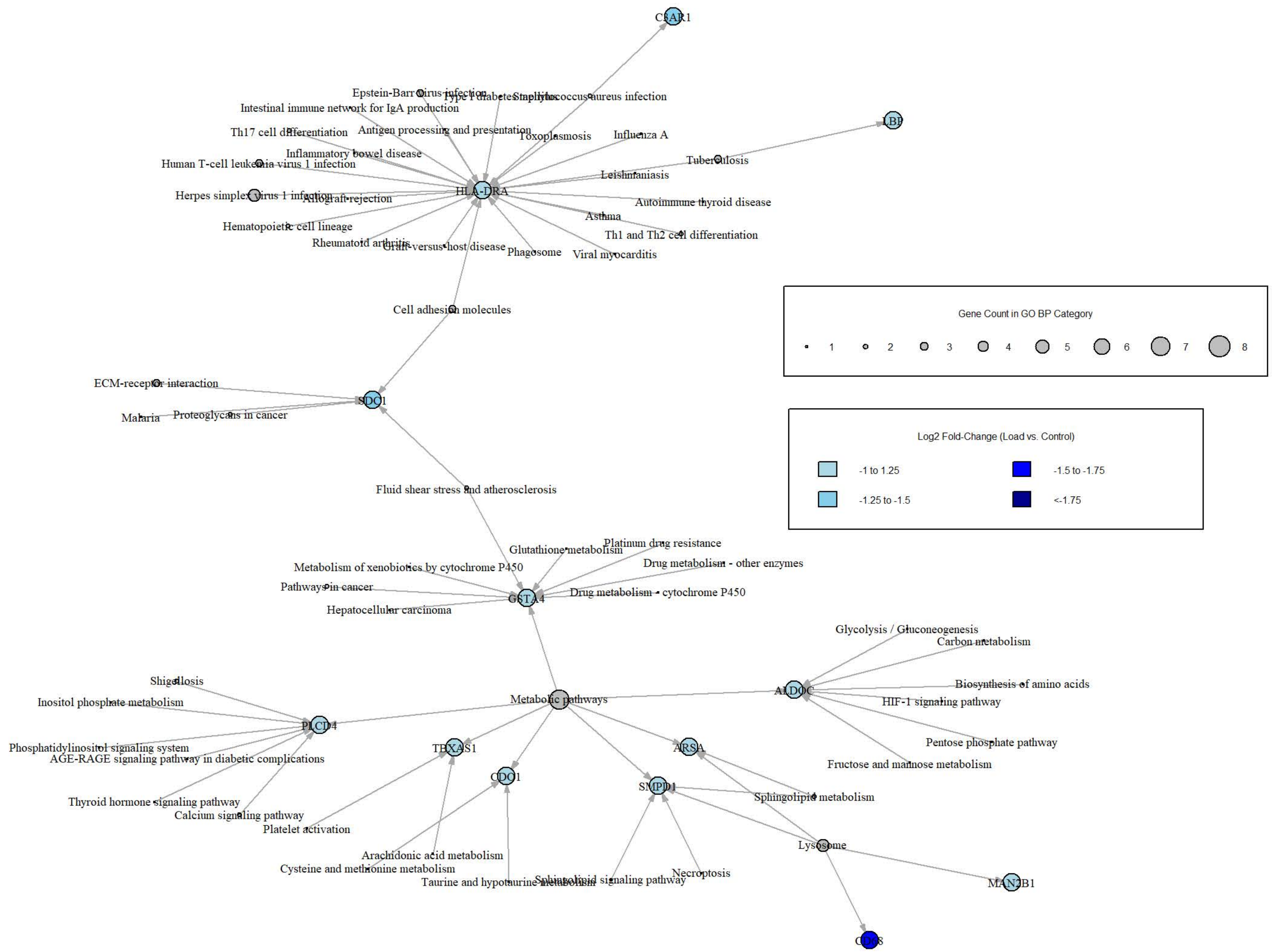

Supplementary Figure 1: Neural graph network illustrating the down-regulated signaling pathways in cultured human growth plate cartilage treated with mechanical loading of 0.4N at 0.77Hz over a duration of 30 seconds across three patients. **(A)** Overview of the neural graph network and **(B)** a magnified view for detailed examination.

(A)

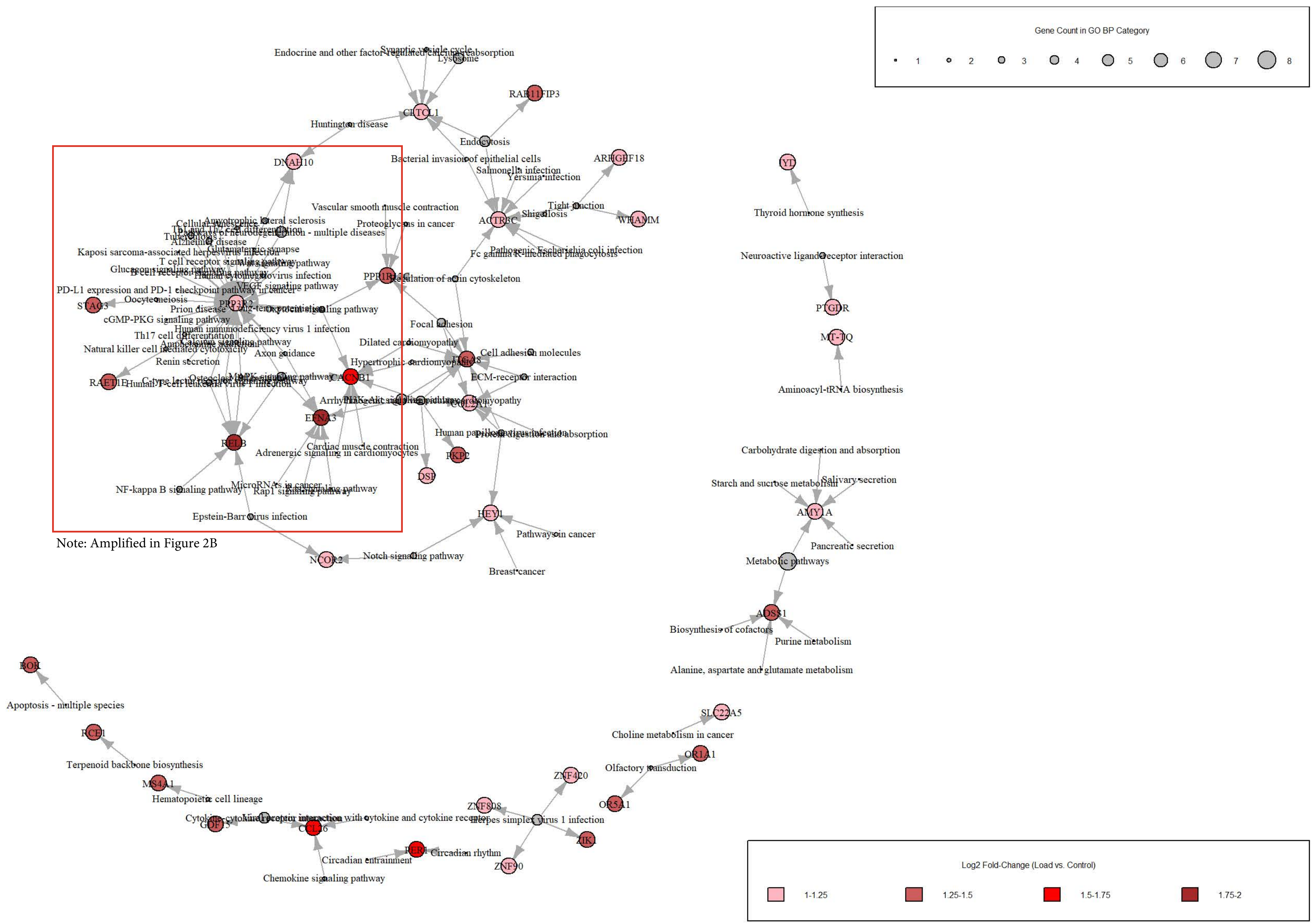

Gene Count in GO BP Category
1
2
3
4
5
6
7
8
Log2 Fold-Change (Load vs. Control)
1-1.25
1.25-1.5
1.5-1.75
1.75-2
Note: Amplified in Figure 2B
Huntington disease
DNAH10
Endocytosis
RAB11FIP3
Bacterial invasion of epithelial cells
Salmonella infection
Yersinia infection
ARHGEF18
Tight junction
WHAMM
Shigellosis
Vascular smooth muscle contraction
Proteoglycans in cancer
Pathogenic Escherichia coli infection
Regulation of actin cytoskeleton
Kaposi sarcoma-associated herpesvirus infection
T cell receptor signaling pathway
VEGF signaling pathway
PD-L1 expression and PD-1 checkpoint pathway in cancer
STAG3
cGMP-PKG signaling pathway
Human immunodeficiency virus 1 infection
Th17 cell differentiation
Natural killer cell mediated cytotoxicity
Renin secretion
Axon guidance
RAET1E
Focal adhesion
Dilated cardiomyopathy
Hypertrophic cardiomyopathy
Cell adhesion molecules
ECM-receptor interaction
Human papillomavirus infection
Protein digestion and absorption
Cardiac muscle contraction
Adrenergic signaling in cardiomyocytes
RELB
DSP
PKP2
NF-kappa B signaling pathway
MicroRNAs in cancer
Rap1 signaling pathway
Epstein-Barr virus infection
HEY1
Pathways in cancer
NCOR2
Notch signaling pathway
Breast cancer
YL
Thyroid hormone synthesis
Neuroactive ligand-receptor interaction
PTGDR
MT-TQ
Aminoacyl-tRNA biosynthesis
Carbohydrate digestion and absorption
Starch and sucrose metabolism
Salivary secretion
Pancreatic secretion
Metabolic pathways
ADSS1
Biosynthesis of cofactors
Purine metabolism
Alanine, aspartate and glutamate metabolism
BOK
Apoptosis - multiple species
RCE1
Terpenoid backbone biosynthesis
MS4A1
Hematopoietic cell lineage
GDF15
Circadian entrainment
PER1
Circadian rhythm
Chemokine signaling pathway
SLC22A5
Choline metabolism in cancer
OR1A1
Olfactory transduction
OR5A1
ZNF420
ZNF808
Herpes simplex virus 1 infection
ZIK
ZNF90

(B)

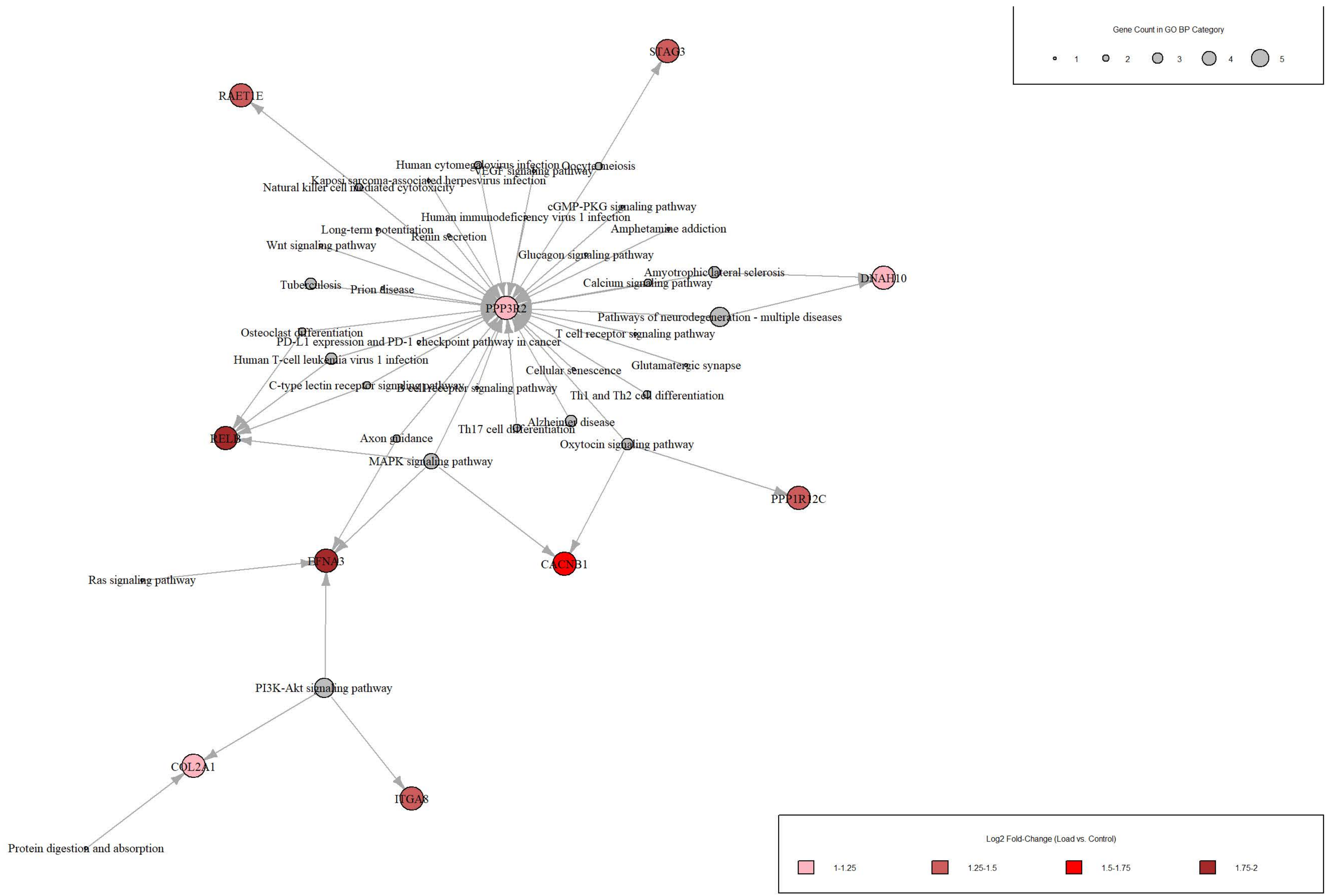

Supplementary Figure 2: Neural graph network depicting the upregulated signaling pathways in cultured human growth plate cartilage subjected to mechanical loading of 0.4N at 0.77Hz over a 30-second duration across three patients. **(A)** Overview of the neural graph network, and **(B)** a magnified view for closer examination.
